# Sonograms of perineal muscles as a noninvasive diagnosis of pyometra and endometritis in beef cows: a preliminary study

**DOI:** 10.5194/aab-68-1-2025

**Published:** 2025-01-06

**Authors:** Amrozi, Sari Yanti Hayanti, Aryogi, Dicky Pamungkas, Dicky Mohammad Dikman, Eko Handiwirawan, Fitra Aji Pamungkas, Herdis, Jakaria, Mokhamad Fakhrul Ulum

**Affiliations:** 1 Division of Reproduction and Obstetrics, School of Veterinary Medicine and Biomedical Sciences, IPB University, Bogor 16680, Indonesia; 2 Research Center for Animal Husbandry, Research Organization for Agriculture and Food, National Research and Innovation Agency of The Republic of Indonesia (BRIN), Bogor 16911, Indonesia; 3 Large Ruminant Standard Testing Station, Ministry of Agriculture, Pasuruan 67184, Indonesia; 4 Department of Animal Production and Technology, Faculty of Animal Science, IPB University, Bogor 16680, Indonesia

## Abstract

Changes in reproductive status affect the perineal muscles. These modifications enable the noninvasive diagnosis of pyometra and mastitis in beef cows. This study aims to assess the thickness and intensity of the coccygeus and levator ani muscles in beef cows affected by the reproductive disorders of pyometra and mastitis. The sample consisted of 59 Bali beef cows, 75 Madura beef cows, and 71 Ongole cross beef cows. The cervical organs, uterine corpus, uterine cornua, and placenta were visualized using transrectal imaging. The resulting images were categorized into two groups: a control group (with nonpregnant nonpartum, pregnant, and nonpregnant postpartum statuses) and a group with reproductive disorders. The coccygeus and levator ani muscles were visualized using transcutaneous imaging. Pregnant Bali beef cows have thicker coccygeus muscles than nonpregnant nonpartum Bali beef cows ( P<0.05 ). Pregnant Madura beef cows have thicker coccygeus muscles than nonpregnant nonpartum and nonpregnant postpartum Madura beef cows ( P<0.05 ). Pregnant Madura beef cows have thicker levator ani muscles than nonpregnant postpartum Madura beef cows ( P<0.05 ). Nonpregnant nonpartum and pregnant Ongole cross beef cows have thicker coccygeus and levator ani muscles than nonpregnant nonpartum and pregnant Bali and Madura beef cows. For pyometra, Bali, Madura, and Ongole cross beef cows have thicker coccygeus and levator ani muscles than endometritis, nonpregnant nonpartum, pregnant and nonpregnant postpartum Bali, Madura, and Ongole cross beef cows. In the control group, only the levator ani muscle of pregnant Bali beef cows had a significant intensity compared to nonpregnant postpartum Bali beef cows ( P<0.05 ). The muscle intensity of the coccygeus and levator ani of nonpregnant nonpartum, pregnant, and nonpregnant postpartum Bali beef cows was higher than nonpregnant nonpartum, pregnant, and nonpregnant postpartum groups for Madura and Ongole cross beef cows. For pyometra, Bali, Madura, and Ongole cross beef cows had higher coccygeus muscle intensity than nonpregnant nonpartum and pregnant Bali, Madura, and Ongole cross beef cows. Reproductive status and disorders affect the diameter of the reproductive tract and plecentome, which affect changes in sonogram thickness and intensity of the coccygeus and levator ani muscles.

## 1 Introduction

Pyometra and endometritis are reproductive ailments affecting female cattle, which have detrimental effects on fertility and productivity, ultimately leading to financial losses in the breeding industry (Deka et al., 2021; El-Roos and Kandiel, 2023). Pyometra is characterized by an increase in the amount of fluid in the form of pus so that the uterus experiences enlargement (Masoumi et al., 2018), while endometrial infections (clinical and subclinical) are characterized by the presence or absence of pus in the uterine lumen without causing changes in the shape of the uterus (Suleymanov et al., 2018). In pyometra, the uterus changes size (caused by an infection that disrupts the work of the prostaglandin hormone), regresses the persistent corpus luteum, and results in high progesterone concentrations so that the cervix closes and pus builds up in the uterine lumen (Masoumi et al., 2018; Sharma et al., 2018; Amin et al., 2021).

Ultrasonography (USG) has been commonly used to detect the dynamics of beef muscle composition without slaughtering (Fiore et al., 2020; Vázquez-Mosquera et al., 2022). USG provides sonogram results in real time (Huang and Zeng, 2017), so it is possible to use USG to diagnose reproductive status based on muscle condition and the results obtained based on real-time imaging (Ulum et al., 2017). A cow's reproductive status and its reproductive organs' conditions affect its body muscles. The study conducted by Lopes et al. (2020) reveals that the reproductive status of cows has an impact on alterations in body muscle composition, particularly in the maintenance of energy balance during pregnancy (McCabe et al., 2021) and postpartum (Megahed et al., 2019). In addition, the application of USG for the perineal muscles (the coccygeus and levator ani muscles) in cows at various stages of pregnancy and postpartum (Ulum et al., 2017; Hayanti et al., 2021) has yielded results that align with the assertion by Budras et al. (2011) about the influence of size on the uterus and about the cow's back muscles.

Research findings regarding the influence of the pregnant and postpartum uterus of cows on the perineal muscles show that it is also possible that reproductive disorders occur in the perineal muscles of cows. Currently, there are no research results regarding the impact of uterine reproductive disorders on the thickness and intensity of perineal muscles. This study aims to measure sonogram thickness and intensity of perineal muscles in beef cows with reproductive disorders of the uterus.

## 2 Material and methods

### 2.1 Study period and location

The study was conducted from 2 November 2019 to 1 February 2020. This study was conducted at the Grati Beef Cattle Research Station, Pasuruan, East Java, Indonesia.

### 2.2 Animals

This study was approved by the Animal Care and Use Committee of the Indonesia Agency for Agricultural Research and Development (number: Balitbangtan/Lolitsapi/Rm/16/2019). The research materials used were 59 Bali beef cows, 75 Madura beef cows, and 71 Ongole cross (PO) beef cows, totaling 205 beef cows. The beef cows consist of nonpregnant nonpartum (NPNP), pregnant (pregnant), nonpregnant postpartum (NPPP), and reproductive disorder (RD) groups. All materials observed were at least 24–168 months old, with reproductive status of having never been pregnant until a maximum of three calving periods. All cows in the body condition score group were on a scale of 2.5–3.5 (scale 5).

### 2.3 Research procedure

The research procedure consisted of two stages: (1) evaluation stage with transrectal ultrasound imaging of the reproductive organs of the cow and (2) transperineal ultrasound imaging of the coccygeus and levator ani muscles of cows. The first and second research stages used the SIUI CTS-900V ultrasound console with a linear probe and frequency of 5.0 MHz. Transrectal and transperineal ultrasound imaging of NPNP cows was performed three times in the first, second, and third months. Transrectal and transperineal ultrasound imaging of pregnant beef cows was performed every month for 1 month, 2 months, and up to 9 months of age. Transrectal and transperineal ultrasound imaging of NPPP (nonpregnant postpartum) beef cows was performed on the 4th and 14th days after parturition. Ultrasound imaging of transrectal and transperineal cows with a RD was performed only once at the time of diagnosis.

### 2.4 Transrectal ultrasound imaging

Transrectal ultrasound imaging of Bali, Madura, and PO beef cows begins with the removal of feces from the rectum to facilitate and expedite the disposition of the ultrasound device, as well as exploration of the position of the reproductive organs. The process was carried out using the left hand, which was protected by a plastic glove and moistened with an ultrasound gel. Next, imaging was performed on the cervical organs, corpus uterine, and cornua uterine using a longitudinal probe (Ulum et al., 2017). The image format for cervical organs, uterine corpus, and uterine cornua in the NPNP, pregnant, and RD groups was a depth of 63 mm and a width of 65 mm. In contrast, the image format for the NPPP group was a depth of 83 mm and a width of 65 mm, and the sonogram file format obtained was Joint Photographic Experts Group (JPEG or JPG).

### 2.5 Transperineal ultrasound imaging

**Figure 1:**
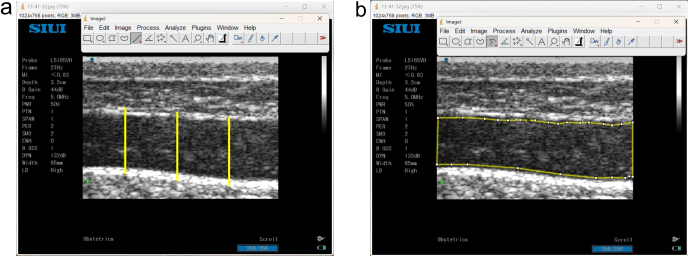
Measurement of muscle thickness and intensity using the ImageJ software (National Institutes of Health, USA). **(a)** Muscle thickness; **(b)** muscle intensity (s = skin, sc = subcutaneous, cm = coccygeus muscle, p = peritoneum).

Transperineal ultrasound was performed transcutaneously in the right perineal area on the coccygeus muscle and levator ani muscle with longitudinal and transverse views (Fig. 1) (Ulum et al., 2017). Transperineal ultrasound imaging begins with wetting and shaving of the hair on the skin surface parallel to the coccygeus and levator ani muscles. The shaved area was about 15 cm × 10 cm, with the remaining hair length of ∼0.3 mm. The shaved area was then gelled, and ultrasound imaging was performed. The image format is 32 mm in depth and 65 mm in width; the sonogram file format obtained is JPG.

### 2.6 Sonogram measurement

Sonograms of the cervix, corpus uterine, and cornua uterine, as well as the coccygeus and levator ani muscles, were obtained using the ImageJ software (National Institutes of Health, USA), without changing the sonogram pixels (1024 × 768). Sonograms of the coccygeus and levator ani muscles were measured for intensity (echogenicity) and diameter. The first step in measuring the intensity and diameter is to set the “set scale” by fixing the “known distance” based on the sonogram depth, which is 32 mm, and the “unit of length” (mm) without changing the sonogram resolution. Muscle thickness was measured using a straight line placed at the outermost point of the muscle fascia and then pulled up to the outermost point of the fascia in the opposite direction to form a straight line. The thickness of each muscle was measured in three different parts, namely, 1/4 , 2/4 , and 3/4 parts, and then the three measurement results were averaged in millimeters (mm). Muscle intensity was measured using a “segmented line”, which was placed at 0.03 mm from the fascia in the muscle and then drawn following the shape of the fascia until the line formed followed the sonogram pattern of the muscle and met at the starting point. The value obtained in the intensity measurement with grayscale is an arbitrary unit (AU). Part of the muscle in the thickness and intensity measurement process is shown in Fig. 1a and b.

### 2.7 Data analysis

All data obtained from the measurements were tabulated using Microsoft Excel 2016. Furthermore, data on the cervix, corpus uterine, and cornua uterine as well as the thickness and intensity of the coccygeus and levator ani muscles of the RD were analyzed using Microsoft Excel 2016. The data obtained from the thickness measurement results and the intensity of the coccygeus and levator ani muscles of Bali, Madura, and PO beef cows with reproductive statuses of NPNP, pregnant, and NPPP were statistically analyzed using SPSS (version 25.0; SPSS Inc., Chicago, IL, USA). The analysis method was the one-way ANOVA test followed by a post hoc Duncan's test, which was used to determine the difference between groups with a significance level of P<0.05 . Data are presented as mean ± standard deviation (SD).

## 3 Results

Transrectal ultrasound results of the NPNP, pregnant, NPPP, and RD of Bali, Madura, and PO beef cows are shown in Table 1.

**Table 1:** Number of Bali, Madura, and PO cows with reproductive statuses NPNP, pregnant, and NPPP as well as pyometra and endometritis as reproductive disorders.

No.	Breed	Reproductive status				
		NPNP	Pregnant	NPPP	RD	
					Pyometra	Endometritis
1	Bali	6	46	3	1	3
2	Madura	7	58	6	1	3
3	PO	3	62	0	1	5
Total		16	166	9	3	11

NPNP represents nonpregnant nonpartum, NPPP represents postpartum nonpregnant, and RD represents reproductive disorder.

### 3.1 Sonogram and diameter of the uterine corpus and cornua of nonpregnant Bali, Madura, and PO beef cows

**Figure 2:**
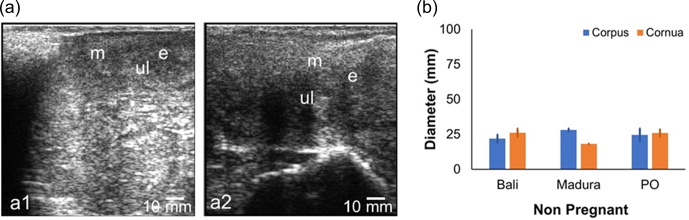
Sonograms **(a)** and diameters **(b)** of the corpus and cornua of nonpregnant nonpartum statuses from the Bali, Madura, and PO cows. **(a1)** Uterine cornua and **(a2)** uterine cornua (e = endometrium, m = myometrium, ul = uterine lumen).

**Figure 3:**
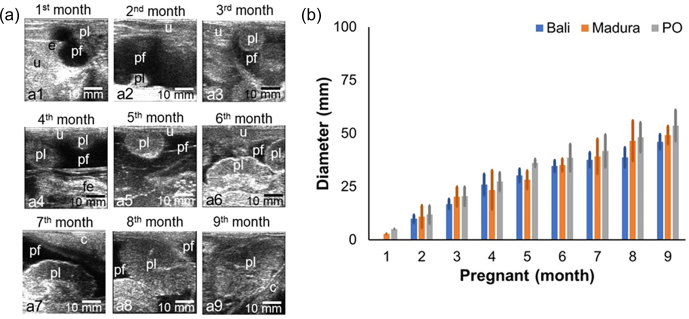
Sonogram and diameter of placentome of Bali, Madura, and PO cows. **(a)** Sonogram placentome; **(b)** diameter of placentome; **(a1)** gestational age 1 month, **(a2)** gestational age 2 months, **(a3)** gestational age 3 months, **(a4)** gestational age 4 months, **(a5)** gestational age 5 months, **(a6)** gestational age 6 months, **(a7)** gestational age 7 months, **(a8)** gestational age 8 months, **(a9)** gestational age 9 months (c = cervix, e = embryo, fe = fetus, pf = placentome fluid, pl = placentome, u = uterus).

**Figure 4:**
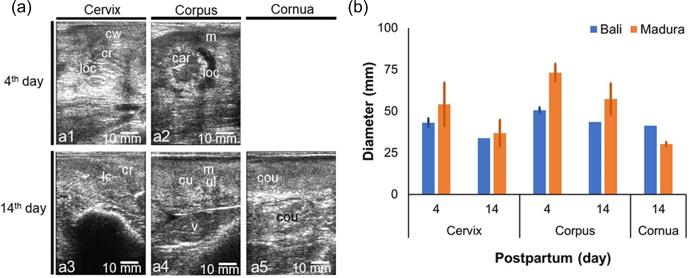
Sonogram **(a)** and diameter **(b)** of cervix, corpus uteri, and cornua uteri postpartum for Bali, Madura, and PO cows. **(a)** Sonogram; **(b)** diameter; **(a1)** cervix day 4, **(a2)** corpus uterus day 4, **(a3)** cervix day 14, **(a4)** corpus day 14, **(a5)** cornua day 14 (car = caruncle, cou = cornua uterus, cr = cervix ring, cu = corpus uterus, cw = cervix wall, loc = lochia, lc = lumen cervix, m = myometrium, v = *vesica urinaria*).

**Figure 5:**
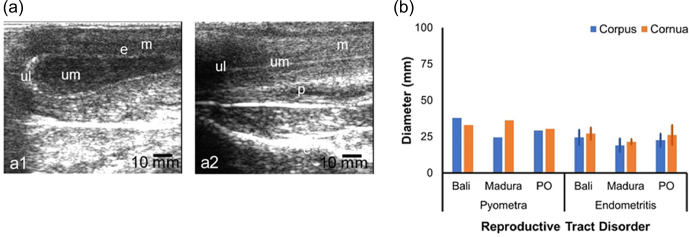
Sonogram **(a)** and diameter **(b)** of the uterine cornua and corpus reproductive disorders in Bali, Madura, and PO cow groups. **(a)** Sonogram; **(b)** diameter; **(a1)** pyometra, **(a2)** endometritis (e = endometrium, m = myometrium, p = peritoneum, ul = uterine lumen, um = uterine mucus).

The NPNP beef cow sonogram showed the structures of the myometrium, endometrium, and uterine lumen (Fig. 2a). The diameter of the reproductive tract for the NPNP group in Bali, Madura, and PO beef cows could be measured accurately (Fig. 2b). Madura cows showed a sonogram of the corpus uteri diameter, which was larger than the corpus uteri diameter of Bali and PO cows (Fig. 2b). The sonogram showed that the diameter of the uterine cornua of Bali cows was larger than those of Madura and PO cows (Fig. 2b). The average uterine diameter of PO beef cows was larger than those of Bali and Madura cows. Sonograms of the corpus and cornua of NPNP beef cows did not show anatomical changes when compared to cows in the pregnant, NPPP, and RD groups (Figs. 2, 3, 4, and 5).

### 3.2 Sonogram and diameter of the beef cow placenta of Bali, Madura, and PO groups

Sonograms of the uteri of Bali, Madura, and pregnant PO cows showed the presence of placenta and amniotic fluid (Fig. 3a). The gestational ages of Bali, Madura, and PO cows with increasing months showed that placental echogenicity became more hypoechoic (Fig. 3a). The placental diameter in PO cows was larger than that in Bali and Madura cows (Fig. 3b). The placentome diameter in Bali, Madura, and PO cows was positively correlated with gestational age (Fig. 3b).

### 3.3 Sonogram and diameter of the postpartum cervix, corpus, and cornua of Bali and Madura beef cows

A sonogram of the NPPP beef cow reproductive tract shows the uterine involution process (Fig. 4). Lochia with low echogenicity (hypoechoic) and caruncle with high echogenicity (hyperechoic) on day 4 of NPPP decreased in size on day 14 (Fig. 4a). The cervix, cornua, and corpus of the uterus decreased in diameter on day 14 compared to the size on day 4 postpartum (Fig. 4b). The diameter of the cervix and corpus uteri of the NPPP Madura cow is larger than that of the NPPP Bali cow. The diameter of the uterine cornua of the NPPP Bali cow was greater than the NPPP Madura cow (Fig. 4b). The diameter of the cornua and uterine corpus of the NPPP cows was larger than that of the NPNP cows (Figs. 2b and 4b).

### 3.4 Sonogram and diameter of reproductive disorders for uterine, corpus, and cornua of Bali, Madura, and PO beef cows

The sonogram of the reproductive tract of the reproductively impaired beef cow also showed changes in the organs (Fig. 5a, b). A sonogram of the uterus with pyometra showed a white line on the lumen wall. Mucus with volume separating the uterine wall as fluid spaces formed in the lumen (Fig. 5a1). The uterus with endometritis showed less mucus and formed a white straight-line appearance in the uterine corpus tract, making the lumen visible (Fig. 5a2). The diameter of the corpus and cornua with pyometra was larger than that with endometritis (Fig. 5b). The diameters of the corpus and cornua uteri of Bali pyometra and endometritis were larger than those of Madura and PO pyometra and endometritis (Fig. 5b). The corpus and cornua of the beef cow uterus with RD had a larger diameter than those of the NPNP beef cow uterus but were smaller than those of the NPPP beef cow uterus (Figs. 1b, 3b, and 5b).

### 3.5 Muscle thickness

**Figure 6:**
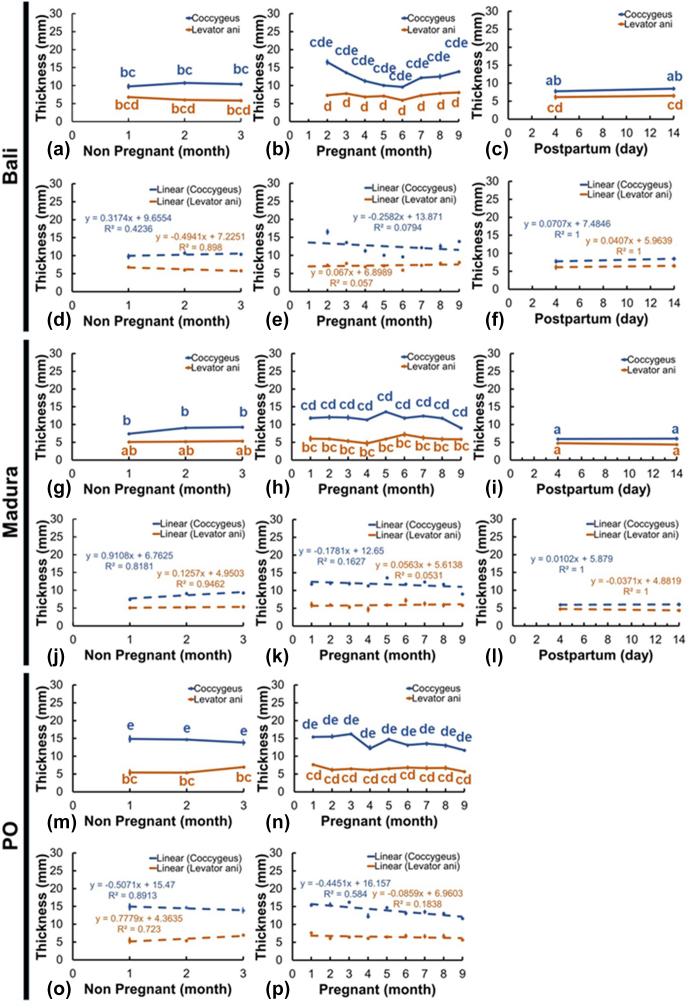
Thickness of the coccygeus and levator ani muscles in Bali, Madura, and PO cows with NPNP, pregnant, and NPPP statuses (**a** – muscle thickness Bali nonpregnant, **b** – muscle thickness Bali pregnant, **c** – muscle thickness Bali postpartum, **d** – linearity of muscle thickness Bali nonpregnant, **e** – linearity of muscle thickness Bali pregnant, **f** – linearity of muscle thickness Bali postpartum, **g** – muscle thickness Madura nonpregnant, **h** – muscle thickness Madura pregnant, **i** – muscle thickness Madura postpartum, **j** – linearity of muscle thickness Madura nonpregnant, **k** – linearity of muscle thickness Madura pregnant, **l** – linearity of muscle thickness Madura postpartum, **m** – muscle thickness PO nonpregnant, **n** – muscle thickness PO pregnant, **o** – linearity of muscle thickness PO nonpregnant, **p** – linearity of muscle thickness PO pregnant).

Thick sonograms of the coccygeus and levator ani muscles showed changes according to changes in reproductive status (Fig. 6a–p). However, not all the changes that occur are significantly different. The thickness of the coccygeus muscle in the Bali NPNP beef cow showed an insignificant change compared to the pregnant and NPPP cows (Fig. 6a–c). Furthermore, the coccygeus muscle thickness of pregnant Bali beef cows showed a significant change ( P<0.05 ) compared to that of NPPP Bali beef cows (Fig. 6b, c). Coccygeus muscle thickness in Madura beef cows showed a significant change ( P<0.05 ) between NPNP, pregnant, and NPPP groups (Fig. 6g–i). Meanwhile, the thickness of the coccygeus muscle in PO NPNP and the pregnant beef cow did not significantly change (Fig. 6m, n).

The thickness of the Bali cow levator ani muscle in NPNP, pregnant, and NPPP groups showed no significant changes (Fig. 6a–c). The thickness of the Madura NPNP levator ani muscle showed insignificant changes compared to those during pregnancy and NPPP (Fig. 6g–i). The thickness of the levator ani muscle of the pregnant Madura group showed a significant change ( P<0.05 ) compared to NPPP (Fig. 6h, i). The thickness of the levator ani muscle of the PO group showed insignificant changes in NPNP reproductive status and pregnancy (Fig. 6m, n).

The results of the ultrasound image of the thickness of the coccygeus and levator ani muscles in Bali, Madura, and PO cows with RD in the form of pyometra showed a thicker muscle than those with endometritis. A comparison of the thickness of the coccygeus and levator ani muscles of Bali, Madura, and PO cows with pyometra and endometritis can be seen in Table 2.

**Table 2:** Comparison of thickness and intensity of ultrasound images on the coccygeus and levator ani muscles of Bali, Madura, and PO beef cow reproductive disorders pyometra and endometritis.

Parameter		Bali		Madura		PO	
		endometritis		endometritis		endometritis	
		Coccygeus	Levator ani	Coccygeus	Levator ani	Coccygeus	Levator ani
Thickness (mm)							
Pyometra	Coccygeus	16.83>15.75	0	15.17>10.53	0	16.79>11.95	0
	Levator ani	0	12.79>11.54	0	6.09>5.52	0	10.08>5.59
Intensity (AU)							
Pyometra	Coccygeus	781.36<924.24	0	779.7>415.34	0	557.94>516.82	0
	Levator ani	0	566.42<691.36	0	458.5>764.95	0	129.62<379.18

mm represents millimeter, AU represents arbitrary unit, > represents larger, and < represents smaller.

### 3.6 Muscle intensity (echogenicity)

**Figure 7:**
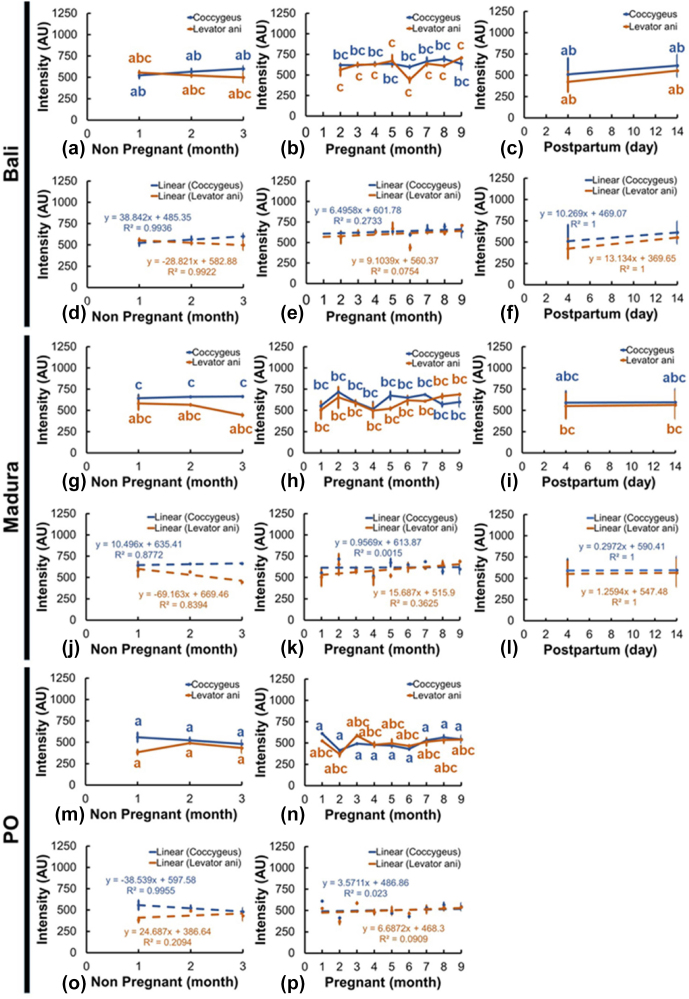
The intensity of the coccygeus and levator ani muscles in Bali, Madura, and PO cows with nonpregnant, pregnant, and postpartum statuses (**a** – muscle intensity Bali nonpregnant, **b** – muscle intensity Bali pregnant, **c** – muscle intensity Bali postpartum, **d** – linearity of muscle intensity Bali nonpregnant, **e** – linearity of muscle intensity Bali pregnant, **f** – linearity of muscle intensity Bali postpartum, **g** – muscle intensity Madura nonpregnant, **h** – muscle intensity Madura pregnant, **i** – muscle intensity Madura postpartum, **j** – linearity of muscle intensity Madura nonpregnant, **k** – linearity of muscle intensity Madura pregnant, **l** – linearity of muscle intensity Madura postpartum, **m** – muscle intensity PO nonpregnant, **n** – muscle intensity PO pregnant, **o** – linearity of muscle intensity PO nonpregnant, **p** – linearity of muscle intensity PO pregnant).

Sonograms of the intensity (echogenicity) of the coccygeus and levator ani muscles showed various changes in different reproductive statuses (Fig. 7a–p). Coccygeus muscle intensity in Bali beef cows showed no significant changes between NPNP, pregnancy, and NPPP reproductive statuses (Fig. 7a–c). The Bali levator ani muscle intensity showed no significant change between the reproductive status of NPNP with pregnancy and NPPP (Fig. 7a–c). Meanwhile, the intensity of the Bali levator ani muscle in the pregnant reproductive status showed a significant change ( P<0.05 ) compared to the NPPP status (Fig. 7b, c). Furthermore, the intensity of the Madura (Fig. 7g–i) and PO (Fig. 7m, n) coccygeus and levator ani muscles in the reproductive statuses of NPNP, pregnant, and NPPP groups showed insignificant changes.

Ultrasound imaging showed that the coccygeus muscle intensity of Madura and PO cows with pyometra cows was hyperechoic compared to that of Madura and endometritis cows. In contrast, the coccygeus muscle intensity of Bali pyometra cows was hypoechoic compared to Bali endometritis beef cows. Ultrasound imaging showed that the intensity of the levator ani muscles of Madura pyometra cows was hyperechoic compared to endometritis Madura cows. In contrast, the intensity of the levator ani muscles of Bali and PO cows with pyometra was hypoechoic compared to those of Bali and PO cows with endometritis. The results of comparing the intensity of the coccygeus and levator ani muscles of Bali, Madura, and PO pyometra and endometritis are presented in Table 2.

## 4 Discussion

The results showed changes in the structure of the uterus in a pregnant beef cow with NPPP and RD compared to NPNP beef cows (Figs. 2–5). The reproductive tract of the NPNP group has a narrower diameter than those of pregnancy, NPPP, and RD groups (Figs. 2–5). However, beef cows with an estrus uterus can also experience a widening in diameter. NPNP cows experienced a widening in diameter of the estrus uterus during estrus until ovulation and decreased after ovulation (Abdelnaby and Abo El-Maaty, 2020). During estrus, the diameter of the uterus can be affected by the presence of intraluminal fluid in the uterine lumen (Ahmadi et al., 2019). The diameter of the uterus during estrus also increases with muscle thickness due to increased blood circulation (Billhaq et al., 2020), which is caused by an increase in hormones in the cow's body (Sugiura et al., 2018). According to the study by De Rensis et al. (2024), the average time from estrus to ovulation is 31.1 h. Therefore, it can be assumed that the uterus with the NPNP group in this study increased in weight, but this occurred in a shorter time than the uterus of the pregnant, NPPP, and chronic RD groups (Figs. 2, 3, 4, and 5).

The diameter of the uterus increases with gestational age (Lin et al., 2021). An increase in gestational age is also followed by an increase in fetal weight (Kouamo, 2018). Fetal weight also has a positive correlation with an increase in placentome diameter (Redifer et al., 2021). The diameter of the placentome of Bali, Madura, and PO cows increased with increasing gestational age (Fig. 3), indicating that increasing the diameter of the placentome in Bali, Madura, and pregnant PO beef cows can be used as a predictor of increased fetal weight. According to Rotheneder et al. (2022), the amnion volume also adjusts to the fetus's needs.

The uterus of NPPP cows undergoes an involution process to start the estrous cycle for the next reproductive period (Elmetwally, 2018). Cornua uterine at the age of 25–30 d postpartum decreases in diameter by up to 3–4 cm from the day of birth (Sheldon et al., 2006). The decrease in uterine diameter was caused by a decrease in the diameter of the caruncle and lochia fluid (Sukareksi et al., 2019), which also appeared on the uterine sonograms of NPPP beef cows. This shows that a decrease in uterine weight is positively correlated with a decrease in uterine diameter. Bali and Madura beef cows experienced a decrease in the diameter of the cornua, uterine corpus, and cervix with increasing involution time (Fig. 4). This indicates that the uteri of Bali and Madura NPPP cows decreased in weight.

Sonogram results of the study showed that the reproductive tract in beef cows with RD showed pyometra and endometritis (Fig. 5a). The diameter of the pyometra uterus was wider than those of the endometrial and NPNP uteri (Figs. 2b and 5b). The pyometra uterus had increased echogenicity of the lumen wall and distension (Tani et al., 2015), which was also observed in this study (Fig. 5a1). A distended pyometra uterus is caused by pus in the lumen (Sharma et al., 2018). In addition to widening the lumen diameter, pyometra also causes widening of the uterine diameter (Melia et al., 2020). Based on the uterine infection category, endometrial uterus in this study was included in the subclinical endometritis category (Salah and Yimer, 2017). Uterine subclinical endometritis occurs when fluid accumulates with less volume so that there is no distension of the uterine lumen (Fig. 4a2). This is in line with the fact that the uterus of cows with subclinical endometritis does not form excess mucus until it exits the reproductive tract (Ricci et al., 2015).

The diameter of the uterus that has increased, especially in pregnant and NPPP beef cows (Figs. 3, 4), influences the size of the abdominal and pelvic spaces of beef cows (Al Masri et al., 2018). The largest coccygeus muscle thickness in NPNP cows was observed in PO cows, followed by Bali and Madura cows, while the largest levator ani muscle thickness was observed in Bali cows, PO cows, and Madura cows (Fig. 6). Coccygeus muscle thickness was the largest in PO cows, followed by Bali and Madura cows, while the thickness of the levator ani muscle in pregnant cows was greater in Bali cows, followed by PO cows and Madura cows (Fig. 7). The thickness of the coccygeus and levator ani muscles in NPPP beef cows was larger in Bali and Madura cows (Fig. 6). Thick coccygeus and levator ani muscles with larger sizes that are dominant in PO and Bali cows may be because PO and Bali cows are a breed of beef cows with a larger body size than Madura cows in the same age group, body condition score, and rearing system (Adinata et al., 2023).

The coccygeus and levator ani muscles in the pelvic area of Bali, Madura, and PO pregnant beef cows generally showed an increase in thickness compared with NPNP beef cows (Fig. 6a, b, g, h, m, and n). However, only the coccygeus muscle in pregnant Madura beef cows showed a significant increase in thickness ( P<0.05 ) compared to the Madura NPNP coccygeus muscle (Fig. 6g, h). The reproductive tract that changes weight causes the coccygeus and levator ani muscles to compensate in the form of weight-bearing activities (Budras et al., 2011), causing muscle thickness to increase. In experimental rats, the muscles and nerves of the coccygeus and levator ani play a role in supporting visceral organs, including reproductive organs (Bremer et al., 2003). The changing size of the pelvic space also causes changes in the muscles of the pelvic area (Hammad et al., 2022). The coccygeus and levator ani muscles play a role in holding uterine organs in the pelvic space (Ulum et al., 2017; Hayanti et al., 2021). It has been shown that vaginal prolapse in rats and sheep occurs because the coccygeus and levator ani muscles in rats and sheep lose their ability to support reproductive tract organs (Mori da Cunha et al., 2021). The height of the maternal pelvis increased during delivery compared to the pelvic space of 8-month pregnant beef cows (Maeda et al., 2022). This is reinforced by the fact that the pelvic area of beef cows differs between cyclical beef cows and early postpartum beef cows (Silva et al., 2019). The equation in these conditions indicates that the muscles and ligaments in the area before parturition are relaxed (Proudfoot, 2019). Relaxin hormone released before delivery causes relaxation of the perineal muscles (Dehghan et al., 2013). This condition causes the coccygeus and levator ani muscles of Bali and Madura NPPP cows to expand such that the muscle size is thinner than that during pregnancy (Fig. 6b, c, h, i). However, in this study, the change in thickness between NPPP and pregnant beef cows occurred significantly ( P<0.05 ) only in the coccygeus muscle of Madura beef cows (Fig. 6h, i). The coccygeus and levator ani muscles in Bali and Madura NPPP beef cows had different thicknesses from those of NPNP cows (Fig. 6a, c, g, i). However, in the Madura group, the coccygeus NPPP muscle showed a significantly thinner muscle size ( P<0.05 ) compared to NPNP (Fig. 6g, i). This indicates that the postpartum coccygeus and levator ani muscles no longer perform uterine load-bearing activities.

Disrupted reproductive organs cause abnormal production of reproductive hormones. Pyometra and endometritis in cows are generally accompanied by the formation of a persistent corpus luteum (Mushonga et al., 2017; Mogheiseh et al., 2019). The level of progesterone in the blood increases and is continuously produced in the ovaries of beef cows with a persistent corpus luteum (Skovorodin et al., 2020). Progesterone levels have also been reported to increase in cows with RDs, such as pyometra and endometritis (Heidari et al., 2019; Amin et al., 2021). The hormone progesterone affects the formation of animal muscle collagen (Nallasamy et al., 2017). This is in line with the results of the study showing that the coccygeus levator ani muscles of Bali, Madura, and PO cows with pyometra were thicker than those with endometritis (Table 1).

The results of this study are also supported by the research by Mair et al. (2020), which showed an increase in muscle fat deposits that affects the increase in muscle thickness due to an increase in sex steroid hormones studied by the human body. Muscle intensity is determined by a combination of the echogenicity of fat and muscle fibers (Gray, 2019). The dynamics of muscle intensity have been reported to be influenced by the number of fat deposits in intramuscular muscles (Nguyen et al., 2021). Dewi et al. (2016) added that the active muscle's ash, protein, and carbohydrate content was higher than the passive muscle content value. At the same time, active muscle fat and water content are lower than those in the passive muscle. Coccygeus and levator ani muscles in PO NPNP and pregnant cows had hypoechoic intensity compared to Bali and Madura cows. Coccygeus and levator ani muscle intensities in NPNP and pregnant Bali cows were hyperechoic compared to Madura cows (Fig. 7). The coccygeus and levator ani muscles of PO cows have hypoechoic intensity compared to those of Bali and Madura cows because the muscle thickness of PO cows is greater than that of Bali and Madura beef cows (Figs. 6 and 7), respectively.

The sonograms of the coccygeus and levator ani muscles generally experienced changes in intensity in Bali, Madura, PO NPNP, pregnant, and NPPP beef cows (Figs. 6, 7). However, only the intensity of the levator ani muscle in pregnant Bali beef cows showed a significant change ( P<0.05 ) compared to NPPP Bali beef cows (Fig. 7b, c). The sonogram intensity of the levator ani muscle in pregnant Bali beef cows was higher than that in the Bali NPPP beef cows. Intramuscular fat is a reserve source of energy (Young et al., 2015). Postpartum beef cows require more energy for the milk production needs of the calves that they give birth to (Gruber et al., 2014). A postpartum beef cow body, if it experiences a limited energy supply, will cause a negative energy balance so that fat reserves in muscle tissue will be used as an energy source (Wang et al., 2019). The Bali beef cow, which requires excess energy during the NPPP period, causes excessive use of excess fat in the muscles, so the intensity of the levator ani muscle is lower than when the beef cow is pregnant (Fig. 7b, c).

The echogenicity of the coccygeus and levator ani muscles in Bali, Madura, and PO cows with pyometra was higher than those with endometritis (Table 1). As previously mentioned, an increase in muscle thickness is caused by an increase in sex steroid hormones, which causes an increase in fat deposits (Mair et al., 2020). The higher echogenicity of the coccygeus and levator ani muscles in pyometra was caused by a greater amount of muscle fat than in endometritis.

## 5 Conclusions

The reproductive statuses of NPNP, NPPP, and RD in Bali, Madura, and PO beef cows were indicated by sonographic changes in the diameter of the cervix, uterine corpus, and uterine cornua. The reproductive status of pregnant Bali, Madura, and PO beef cows was indicated by sonographic changes in placental diameter. Changes in the thickness and intensity of the coccygeus and levator ani muscles were affected by changes in the reproductive status of the Bali, Madura, and PO beef cows. This research needs to be conducted more deeply based on the body size of each breed of beef cow to determine changes in muscle thickness and intensity, which can be used as a method for diagnosing reproductive status or reproductive disorders.

## Data Availability

Some of the data utilized in this paper were derived from Sari Yanti Hayanti's postgraduate thesis conducted during her studies in the Reproductive Biology program at the Postgraduate School of IPB University. The thesis data are archived in the IPB University repository and are accessible online at https://repository.ipb.ac.id/handle/123456789/105939 (Hayanti, 2024).
